# An integrated clinical approach to children at genetic risk for neurodevelopmental and psychiatric conditions: interdisciplinary collaboration and research infrastructure

**DOI:** 10.1186/s11689-024-09552-x

**Published:** 2024-07-05

**Authors:** Jane Summers, Danielle Baribeau, Polina Perlman, Ny Hoang, Sunny Cui, Aneta Krakowski, Patricia Ambrozewicz, Ariel Ho, Thanuja Selvanayagam, Kinga A. Sándor-Bajusz, Katrina Palad, Nishi Patel, Sarah McGaughey, Louise Gallagher, Stephen W. Scherer, Peter Szatmari, Jacob Vorstman

**Affiliations:** 1 Department of Psychiatry, Temerty Faculty of Medicine, University of Toronto, Toronto, ON Canada; 2 Program in Genetics and Genome Biology, The Hospital for Sick Children, Toronto, ON Canada; 3 Department of Psychiatry, The Hospital for Sick Children, Toronto, ON Canada; 4 Holland Bloorview Kids Rehabilitation Hospital, Toronto, ON Canada; 5 Department of Genetic Counselling, The Hospital for Sick Children, Toronto, ON Canada; 6 The Centre for Applied Genomics, The Hospital for Sick Children, Toronto, ON Canada; 7 Centre for Addiction and Mental Health, Toronto, ON Canada; 8 McLaughlin Centre and Department of Molecular Genetics, University of Toronto, Toronto, ON Canada; 9 Department of Molecular Genetics, University of Toronto, Toronto, ON Canada; 10 Autism Research Unit, The Hospital for Sick Children, Toronto, ON Canada; 11 Hospital for Sick Children, Peter Gilgan Centre for Research and Learning, 686 Bay Street Room 12.9702, Toronto, ON M5G 0A4 Canada

**Keywords:** Interdisciplinary clinic, Psychiatry, Psychology, Genetics, Neurodevelopmental disorder, Mental health, Genetic disorder, Research framework

## Abstract

**Background:**

A sizeable proportion of pathogenic genetic variants identified in young children tested for congenital differences are associated with neurodevelopmental psychiatric disorders (NPD). In this growing group, a genetic diagnosis often precedes the emergence of diagnosable developmental concerns. Here, we describe DAGSY (Developmental Assessment of Genetically Susceptible Youth), a novel interdisciplinary ‘genetic-diagnosis-first’ clinic integrating psychiatric, psychological and genetic expertise, and report our first observations and feedback from families and referring clinicians.

**Methods:**

We retrieved data on referral sources and indications, genetic and NPD diagnoses and recommendations for children seen at DAGSY between 2018 and 2022. Through a survey, we obtained feedback from twenty families and eleven referring clinicians.

**Results:**

159 children (mean age 10.2 years, 57.2% males) completed an interdisciplinary (psychiatry, psychology, genetic counselling) DAGSY assessment during this period. Of these, 69.8% had a pathogenic microdeletion or microduplication, 21.5% a sequence-level variant, 4.4% a chromosomal disorder, and 4.4% a variant of unknown significance with emerging evidence of pathogenicity. One in four children did not have a prior NPD diagnosis, and referral to DAGSY was motivated by their genetic vulnerability alone. Following assessment, 76.7% received at least one new NPD diagnosis, most frequently intellectual disability (24.5%), anxiety (20.7%), autism spectrum (18.9%) and specific learning (16.4%) disorder. Both families and clinicians responding to our survey expressed satisfaction, but also highlighted some areas for potential improvement.

**Conclusions:**

DAGSY addresses an unmet clinical need for children identified with genetic variants that confer increased vulnerability for NPD and provides a crucial platform for research in this area. DAGSY can serve as a model for interdisciplinary clinics integrating child psychiatry, psychology and genetics, addressing both clinical and research needs for this emerging population.

**Supplementary Information:**

The online version contains supplementary material available at 10.1186/s11689-024-09552-x.

## Background

High-resolution genome-wide technologies are increasingly considered routine part of the diagnostic workup for a range of disorders [1, 2]. With decreased costs and growing diagnostic yields, we can now identify a causal/contributory pathogenic genetic variant in ∼ 10–20% of autistic individuals, 15% of children with epilepsy, up to 50% of those with intellectual disability, and up to 80% of children with complex multisystem conditions [1–4].

However, the majority of human genes are pleiotropic, i.e., the same gene can be involved in more than one biological function [5, 6]. Of relevance to mental health, approximately 80% of human genes are brain-expressed [7], and rare genetic variants in over 1,000 genes have been directly or loosely associated with neuropsychiatric and/or neurodevelopmental impacts [8]. Henceforth, the term neurodevelopmental psychiatric disorders (NPDs) will refer to a broad group of phenotypes including intellectual or learning disability (ID and LD), autism spectrum disorder (ASD), ADHD, as well as mood, anxiety, psychotic and other psychiatric disorders^1^.

Historically, in patients without medical complexities, clinical genetic testing is typically considered *following* the diagnosis of an NPD, such as ASD or ID [10, 11], known as a *phenotype-first* approach [12]. Establishing a genetic basis for NPD may offer an etiological explanation to families and help connect them to genetic condition-specific resources and communities of support [13], as well as inform genetic counseling [14]. Here, the role of a genetic diagnosis is to provide an explanation for the observed NPD phenotype. Knowledge of the underlying genetic etiology can in some cases also provide specific treatment guidance, for example in the case of sleep disturbance in Kleefstra syndrome [15] or the use Clozapine in individuals with 22q11.2 deletion syndrome [16]. Increasingly, biological insights inferred from genetic studies inform clinical trials for individuals with NPDs associated with specific genetic variants [17–19]. Examples of the latter include studies examining the effects of insulin-like growth factor – 1 (IGF-1) in individuals with variants in *SHANK3* (Phelan-McDermid syndrome) [20] or in *MECP2* (Rett Syndrome) [21]; of Rapamycin analogues for NPDs related to *TSC1/TSC2* variants (Tuberous Sclerosis Complex) [22] or related to *PTEN* variants [23]; Arbaclofen in NPDs related to *FMR1* variants (Fragile X) [24]. Further building on the foundation of recognizable specific genetic etiologies, other studies examine the feasibility of novel therapeutic strategies aimed at correcting the genetic defect at the DNA or RNA level [25, 26].

There is a growing list of genetic conditions associated with increased rates of NPD phenotypes such as ASD, ADHD, ID, and psychosis. Increasingly, these genetic diagnoses are made early in life prior to the emergence of psychopathology. For some relatively well established conditions, the recognition of the genetic etiology has promoted the development of clinical practice recommendations which include strategies to monitor the possible emergence of NPD symptoms, for example for 22q11.2 deletion syndrome (22q11DS) [27], Williams syndrome [28], Prader-Willi syndrome [29], Fragile X syndrome [30] and Tuberous Sclerosis Complex [31]. Note that in this scenario, an early genetic diagnosis implies a future vulnerability for NPD, exemplifying a *genotype-first* approach.

Up until recently, this concept mainly referred to genetic testing without an a priori specific diagnostic suspicion [32]. However, given the increased uptake of genetic testing very early in life, the notion can be further expanded to refer to any situation where a genetic diagnosis precedes and informs clinical management. Prominent examples of the latter include preventive interventions for carriers of pathogenic variants in *BRCA1* or *BRCA2* associated with breast cancer [33], in *APC* associated with colon cancer [34] or in several genes associated with cardiomyopathies and arrhythmias [35]. In recent years, it is increasingly recognized that this type of genotype-first scenario has also entered the domain of mental health, spurred by the gradual increase in children and youth identified as “at risk for NPD” based on the presence of a pathogenic genetic variant [36]. This is particularly poignant when a genetic diagnosis, triggered by congenital difference or illness, is made in a newborn and precedes the emergence of developmental or behavioral concerns by years. The early recognition of such genetically mediated vulnerability for NPDs can induce anxiety in caregivers, creating an imperative to provide appropriate guidance and clinical support for patients and their families as well as an opportunity to explore preventive mental health strategies.

Importantly, these considerations are not merely theoretical. We have recently shown that in 5–10% of clinical genetic tests ordered for a physical or congenital indication, and in 52% of those with a positive diagnostic result, the identified (likely) pathogenic variant was also associated with possible NPD outcomes [37]. Regardless of whether the genetic diagnosis precedes or follows the emergence of NPD symptoms, children with genetically mediated vulnerability for NPD outcomes are a growing phenomenon for clinicians working in psychiatry, psychology, neurology or developmental pediatrics. The increasing identification, through early genetic testing, of young children with increased vulnerability for various NPD outcomes introduces a novel “genetic-diagnosis-first” clinical scenario in the field of mental health and represents a thus far largely unmet clinical need for affected families. Concomitant with the growing uptake of genetic testing early in life and the increasing yield of these tests, there is an urgent need for a better understanding of the variable and often pleiotropic phenotypic expression of NPD-related pathogenic variants, and subsequently, their optimal treatment strategies. The rare occurrence of many of these variants forms a daunting challenge to the research community.

DAGSY (Developmental Assessment for Genetically Susceptible Youth) Clinic is a new clinical service model that integrates child psychiatry, psychology and genetics in an attempt to address the clinical and research needs of this novel fast-emerging patient population. The aims of this manuscript are to provide a rationale for and a procedural description of DAGSY; to report on our observations in the first 159 patients; and to report on feedback from families and referring clinicians.

## Methods

### Overview

DAGSY was initiated at The Hospital for Sick Children (SickKids), a large pediatric teaching hospital in Toronto, Canada. Its development, in line with the hospital-wide Precision Child Health Initiative and the SickKids Genome Clinic [38], was designed to specifically address the unmet clinical need of children with a genetic disorder associated with NPD, by providing interdisciplinary psychological, developmental and psychiatric evaluations informed by clinical knowledge available for each specific pathogenic variant: effectively, a “genetic-diagnosis-first” clinical psychiatric and developmental consultation service. In addition, we implemented a standardized assessment protocol to facilitate the potential use of clinical data for research purposes. DAGSY is a full clinical service with regular clinical billing entirely covered by the government-funded health care plan. We collected referral data, diagnostic conclusions and recommendations provided for the first 159 children evaluated in the DAGSY Clinic, and results of family and clinician surveys designed to assess the perceived benefits of the DAGSY evaluation and satisfaction with the clinical process, along with recommendations for future studies.

### Retrospective review of DAGSY reports

We reviewed all patients who completed a comprehensive DAGSY assessment between March 2018 and December 2022, which was authorized as a Quality Improvement evaluation. We extracted demographic data (sex and age at assessment), referral source and reasons(s) for referral, genetic test information (type of test and result), pre-existing and new neurodevelopmental and psychiatric diagnoses and recommendations provided to parents and clinicians. Study data were collected and managed using REDCap electronic data capture tools hosted at The Hospital for Sick Children [39, 40]. REDCap (Research Electronic Data Capture) is a secure, web-based software platform designed to support data capture for research studies, providing (1) an intuitive interface for validated data capture; (2) audit trails for tracking data manipulation and export procedures; (3) automated export procedures for seamless data downloads to common statistical packages; and (4) procedures for data integration and interoperability with external sources. Supplement 1 (available online) provides an overview of standardized measures implemented in the DAGSY assessment.

### Parent and clinician survey

For DAGSY assessments that were completed in 2021–2022 (*n* = 61), families and referring clinicians were sent an electronic link to a survey via REDCap (see Supplement 2 available online for survey items). Participation in the survey was voluntary and responses were provided anonymously. Survey responses were captured using a Likert-type scale that provided five response options (ranging from *strongly disagree* to *strongly agree*) and assessed perceptions regarding the DAGSY assessment and satisfaction with the clinical process. Dichotomous ratings (yes/no) were obtained for a subset of questions. The survey was approved by the Research Ethics Board at The Hospital for Sick Children.

### Data analysis

Descriptive statistics, including means, medians, standard deviations and ranges, were obtained for continuous data. Frequencies and percentages were obtained for non-continuous (categorical) data. Data were exported from the REDCap database to IBM SPSS Statistics for MacIntosh (Version 28.0.1.1) for analysis.

## Results

### Program description

**Fig. 1:**
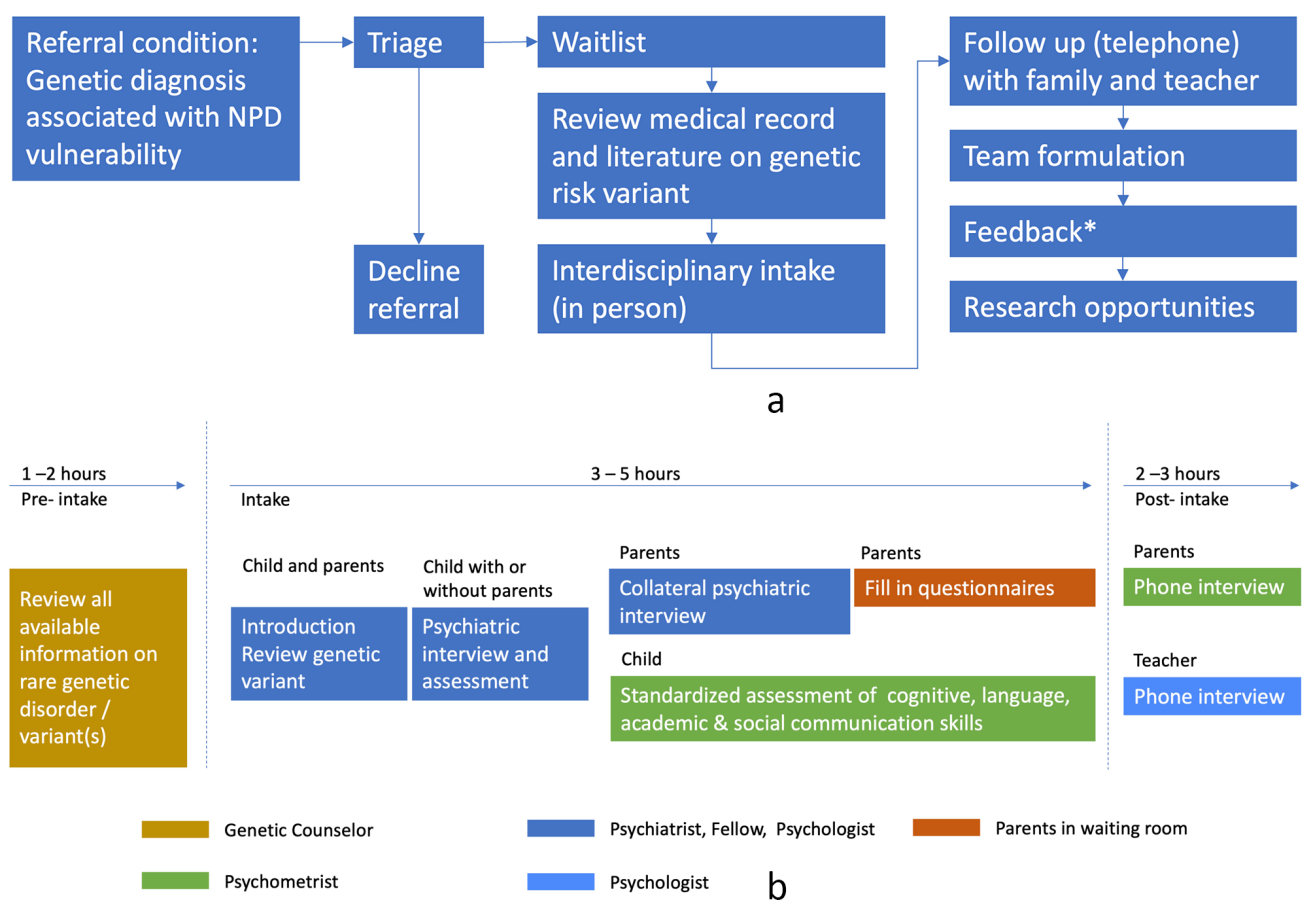
(**a**) Overview of DAGSY referral flow. (**b**) Assessment procedure for DAGSY *Feedback appointment initially in person, during pandemic exclusively online, currently offered in both modalities to families

DAGSY is a clinical service for children between 12 months and 18 years. While for most current psychiatry clinics, behavioral symptoms or concerns are a condition for referral, DAGSY accepts referrals regardless of the presence or suspicion of symptoms, as long as the child is identified to have a molecularly confirmed genetic variant associated with NPD *risk*. To determine NPD association, published data and genomic databases are reviewed (e.g., OMIM, GeneReviews, ClinGen). Variants associated with known disorders where manifestation of neurodevelopmental or psychiatric symptoms are part of the phenotype are designated as having NPD implications. For rarer variants/genes, case reports and clinical catalogs (e.g., ClinVar) are reviewed to determine evidence of NPD association [37]. Consequently, the first step in the DAGSY clinic process (see Fig. 1a), triage, involves a genetic counselor on the DAGSY team carefully reviewing the patient’s genetic test results and evaluating their association with NPD.

The rationale for DAGSY is predicated on genetic vulnerability for NPD, which typically is heterogenous (i.e., cuts across different phenotypic domains) as well as variably penetrant (i.e., with variable risk effect sizes for each phenotype) for most pathogenic variants [41]. As a result, most children can be considered at risk for symptoms across cognitive, academic and behavioral/psychiatric domains. To address this heterogeneity in a comprehensive manner, evaluations at DAGSY are conducted by an interdisciplinary team including a child and adolescent psychiatrist, a child psychiatry fellow, a psychologist and a psychometrist, as well as a genetic counselor. The interdisciplinary nature of clinical evaluations lends itself to an efficient organisation, such that both psychiatric evaluation and standardized cognitive and academic assessment can be completed in a single in-person session (see Fig. 1b). A more detailed description of the DAGSY process is provided in the online supplement (See Supplements 1 and 3).

### DAGSY clinical findings 2018–2022

Table 1 shows demographic information, referral reason and source and genetic diagnosis for 159 children completing an assessment in the DAGSY Clinic between March 2018 and December 2022.

**Table 1:** Demographic and referral details

	**Entire sample**	**Age groups (** ***n*** **)**		
		**0–5**	**6–10**	**11+**
Number Sex - % Male Age in years	159 57.2	16	80	63
Mean SD Range	10.2 3.8 0.92–19.1			
	**Entire sample (** ***n*** **, %)**	**Age groups (** ***n*** **)**		
		**0–5**	**6–10**	**11+**
**Referral Source**				
*Internal* Genetics + syndrome clinics Psychiatry + Psychology Other specialties and units Pediatrics Neurology *External* Pediatrics Developmental Pediatrics Genetics GP, Psychiatry, Neurology	*132 (83)* 104 (65.4) 9 (5.7) 8 (5.0) 7 (4.4) 4 (2.5) *27 (17)* 10 (6.2) 8 (5) 6 (3.8) 3 (1.9)	*13* 13 0 0 0 0 *3* 2 0 1 0	*63* 51 3 4 3 2 *17* 5 6 4 2	*56* 40 6 4 4 2 *7* 3 2 1 1
**Referral Reason** ^**a**^				
Comprehensive Psychiatric Behavior Medication Learning	75 (47.2) 58 (36.5) 37 (23.3) 17 (10.7) 16 (10.1)	8 4 5 1 2	37 25 20 8 10	30 29 12 8 4
**Genetic Diagnosis**				
Deletion or duplication Single gene variant Chromosomal disorder VUS^b^	111 (69.8) 34 (21.4) 7 (4.4) 7 (4.4)	14 2 0 0	51 18 5 6	46 14 2 1

^a^ More than one reason could be specified at referral

^b^ Variant of Uncertain Significance

Males were referred to the clinic more often (57.2%) and the mean assessment age for all children was 10.2 years. Most referrals were from specialties and clinics within the hospital, most notably clinical genetics and genetic syndrome clinics. Children were referred most often for a comprehensive (developmental and mental health) assessment. The majority of children assessed in DAGSY had a pathogenic microdeletion or microduplication. A small number of children with a Variant of Uncertain Significance (VUS) were accepted to DAGSY if emerging evidence suggested pathogenicity and the phenotypic consequences were thought to likely include NPD risk. Examples of genetic diagnoses are provided in Table 2.

**Table 2:** Examples of genetic risk variants for children assessed in DAGSY

Category	Examples
Microdeletion and microduplication disorders	• 22q11.2 • 16p11.2 • 17p11.2 • 5p • 9p • 3q13 • 12q
Single gene disorders	• *ARID2* • *ARID1B* • *ANKRD11* • *SLC6A8* • *PTCHD1* • *SHANK3* • *K1F1A* • *EHMT1*
Chromosomal disorders	• 47XXX • 47XXY • 45X • Trisomy 21

### Pre-existing and new clinical diagnoses

**Fig. 2:**
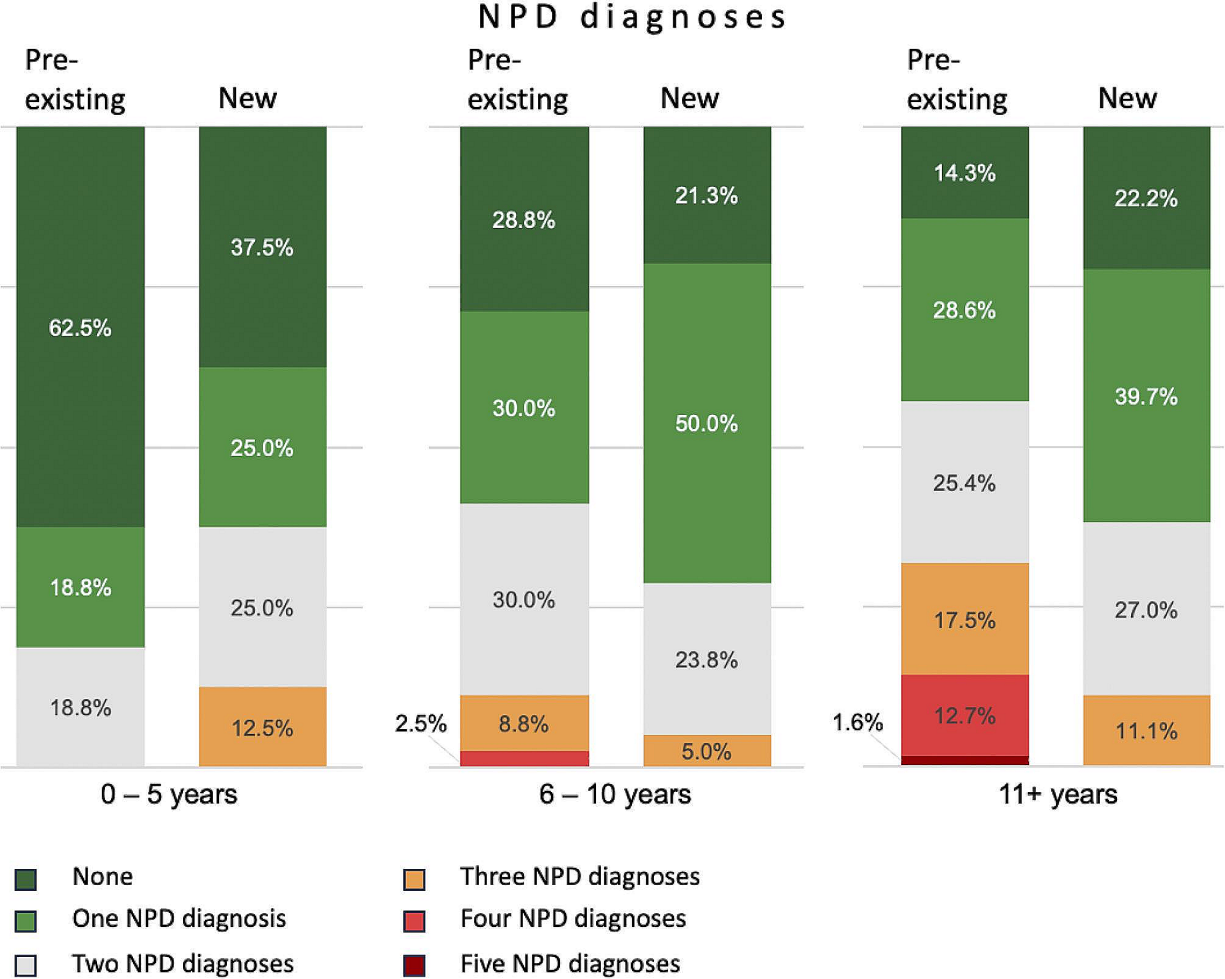
Pre-existing and new NPD diagnoses prior to and following DAGSY assessment This figure shows the proportion of children per NPD diagnosis count (including developmental delay as a diagnosis), calculated for three age groups. The proportion of children without a pre-existing NPD diagnosis is highest in the youngest age group, whereas in the older age groups the proportion of children with multiple NPD diagnoses increases, indicative of an accumulation of diagnoses over time in this population

Approximately 1 in 4 of children (42/159 or 26.4%) did not have any NPD diagnosis at referral to DAGSY. This proportion was even larger, approximately 1 in 3 (33.8%) when including children with developmental delay as the only diagnosis at referral. Since the inception of DAGSY, the proportion of children referred based on genetic risk alone (i.e., without an existing NPD diagnosis) showed an increasing trend (15.3% in 2018; 27.5% in 2019; 29.3% in 2020; and 31.5% in 2021). Figure 2 shows NPD diagnoses at the time of referral to DAGSY as well as new diagnoses made following DAGSY assessment.

Pre-existing NPD diagnoses consisted of ADHD (44/159 or 27.7%), ASD (27%), ID (25.2%), global or developmental delay (18.9%), learning disability (12.6%), language or speech disorder (10.1%), anxiety disorder (5.6%), oppositional defiant disorder (2.5%), tics (1.9%), fetal alcohol spectrum and obsessive compulsive disorder (1.3% each) and psychosis, depression, adjustment, sleep, developmental coordination disorder or communication disorder (0.6% each; note that many children had more than one diagnosis at referral).

Following the DAGSY interdisciplinary assessment, 122 of 159 children (76.7%) were diagnosed with a new NPD, consisting of ID (24.5%), anxiety disorder (20.7%), ASD (18.9%), learning disorder (16.4%), ADHD (8.8%), language or speech disorder (6.9%), unspecified NDD, disruptive behavior disorder and global or developmental delay (2.5% each), excoriation disorder, psychosis, eating disorder, oppositional defiant or intermittent explosive disorder (1.3% each), catatonia, bipolar I or adjustment disorder (0.6% each).

Of those children with a new diagnosis of ID (*n* = 39), in 23 (59%) the newly diagnosed ID represented a change from a previous diagnosis of global or developmental delay and 4 (10.3%) were a change from a previous diagnosis of learning disorder. Other diagnoses that were removed consisted of ADHD (1 child), ID (5 children) and language or speech delay (4 children). The mean age for a new diagnosis of ID was 11.3 years (SD = 3.4 years, Range = 2.1–16.9 years), and for ASD this was 10.0 years (SD = 3.7 years, Range = 2.0-16.8 years).

### Recommendations to parents

The most common recommendation provided following DAGSY assessment involved home or school-based strategies (117/159 = 73.6%). For parents, these included concrete strategies such as establishing predictable routines, using proactive approaches for managing transitions, and dealing with outbursts and teaching functional communication and self-help skills. For schools, these included tailoring academic demands to fit children’s skills and abilities and consideration of special education placement and services. Programs and services were recommended in 67.9% of cases; these included community-based programs for children with ASD or ID, mental health, or respite services and parent training for the management of challenging behavior. Follow-up psychoeducational assessments or psychiatric re-assessment as part of disorder-specific surveillance (e.g., risk of psychosis in youth with 22q11DS) and/or additional assessments (e.g., sensory processing, functional behavioral assessment) were recommended in 56.6% of cases. Therapies (e.g., behavior therapy or applied behavior analysis, cognitive behavior therapy, occupational or communication therapy) were recommended in 51.6% of cases. Resources and information (e.g., links to disorder-specific support groups for parents, lay summaries of learning needs and educational strategies for teachers, websites for government-funded programs) were provided in 49.7% of cases. Medication recommendations were made in 44% (e.g., for initiating, switching, or altering the dose of psychotropic medications), and recommendations for parent support (e.g., social work, case coordination) were provided in 18.2% of cases. The median number of recommendations provided per family was 3.5 (range 1–7).

### DAGSY as a platform for clinical research

The assessments at DAGSY follow a standardized protocol (see Supplement 1) such that observations are not only useable for the primary clinical purposes, but also for research if needed. Ethical approval was obtained to discuss research opportunities with parents after completion of the clinical process. This approach, combined with a steady stream of patients with often exceedingly rare genetic conditions creates a versatile platform for clinical research. The following are examples of research output made possible by DAGSY; (1) participation in the Genes to Mental Health Network (G2MH), an NIMH-funded initiative that amongst others collect genotype and phenotype data in individuals with copy number variants at 16p11.2 or 22q11.2 [8, 42]; (2) a case report on a 16p13.3 deletion [43]; (3) a case report describing treatment in a patient with a rare genetic variant, post gene therapy [44]; (4) a case series describing the phenotypic profile of 11 individual carriers of variants in the same gene, suggesting for the first time its potential pathogenicity (the first two patients of this series were identified at DAGSY); (5) participation in a multi-site industry-sponsored clinical trial (NCT05290493) (manuscripts for the latter two are in preparation). In addition to these studies directly related to patients, the clinical experience at DAGSY has inspired a review on the psychological support for caregivers following a genetic diagnosis with impact on neurodevelopmental risk [45]. Finally, the stream of patients with specific variants fosters the building of dedicated patient cohorts. Current DAGSY efforts include the collection of data of patients with *NF1* variants, *NRXN1* deletions and 22q11.2 deletions, which in turn improves the prospects of successful grant funding and international collaborations.

### Family survey results

**Fig. 3:**
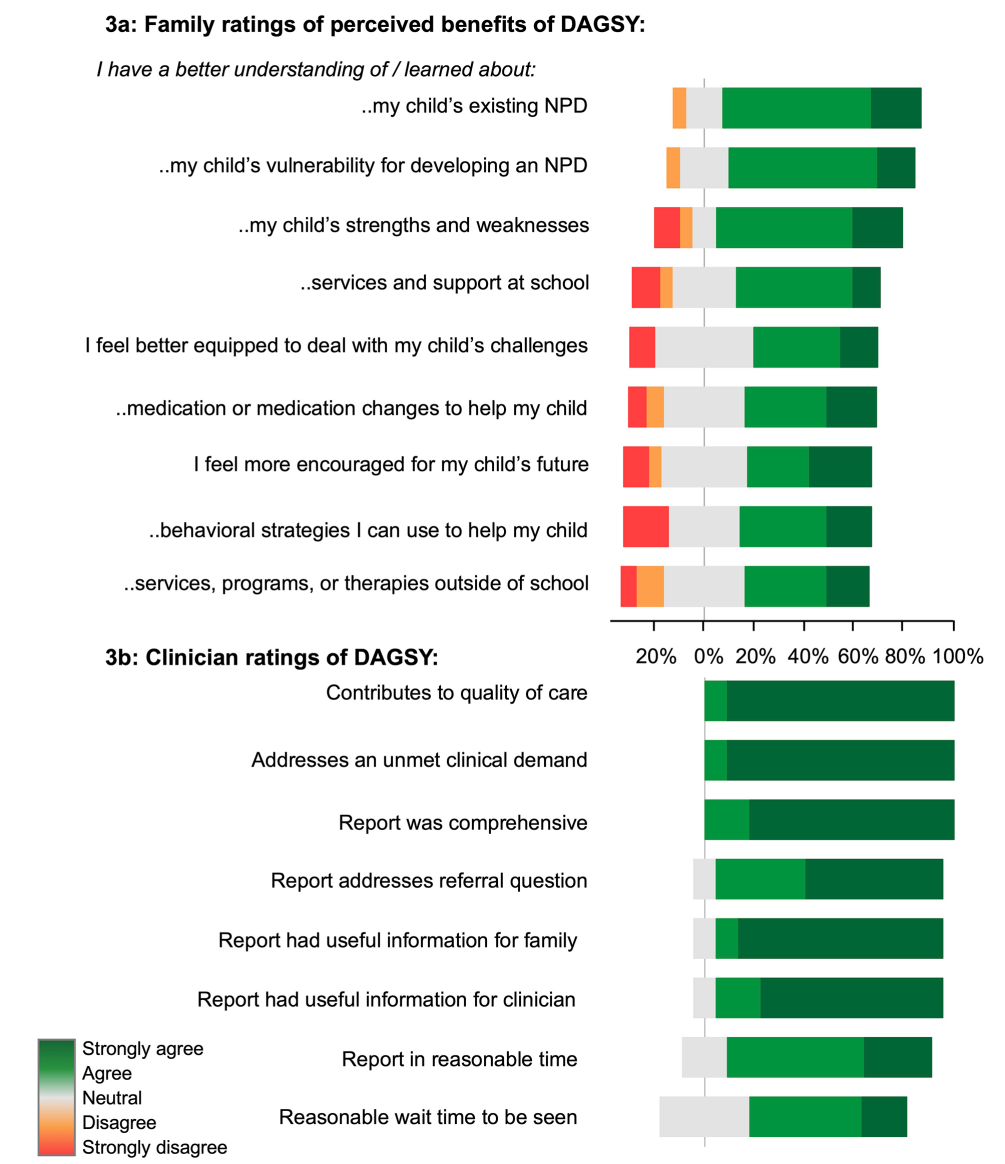
Survey results

Surveys were returned by 26 of 61 families who agreed to participate after being sent a REDCap link (42.6% response rate); six families were excluded from the analysis because they only answered the demographic questions. Mothers completed surveys 70.8% of the time, fathers 16.7% and other (e.g., grandparent) 12.5%. The age of respondents was less than 34 years for 19%, between 35 and 50 years for 66.7% and 51 or more years for 14.3%. The education level of respondents was 8.7% for high school, 69.6% for college or university and 21.7% for graduate school. At the time of survey completion, the last interaction with DAGSY was less than 6 months for 55.6% of respondents, between 6 and 12 months for 27.8%, greater than 12 months for 11.0%; 5.6% were unable to remember. Parent needs prior to the DAGSY evaluation included assessment of their child’s behavior, development and learning abilities, information about their prognosis and recommendations for resources and supports. The majority of families (90%) were satisfied with information provided by DAGSY in relation to their needs. Perceptions of the DAGSY evaluation were generally positive (see Fig. 3a). The highest satisfaction rates were in relation to families gaining a better understanding of their child’s NPD and genetic vulnerability for developing NPD, as well as having a better understanding of their child’s strengths and weaknesses. Areas for further improvement related to learning about school services and therapies outside of school, and insight into pharmacological or behavioral strategies to help their child. One-half of parents reported feeling more encouraged about their child’s future and better equipped to manage their child’s challenges upon visiting DAGSY.

In addition, most families (85%) reported trying to implement recommendations from the report. Suggestions for improvement included having DAGSY staff make direct referrals for programs and services, providing follow-up to the extent possible, expanding the program to help more families and promoting DAGSY within the wider healthcare community.

Families also expressed satisfaction regarding their experience with and the quality of care provided by the DAGSY clinic (80–90% agree or strongly agree), as well as with specific procedural aspects including the initial intake and feedback meetings and information in the written report.

### Clinician survey results

Surveys were returned by 15 of 19 referring clinicians who agreed to participate after receiving a REDCap link (78.9% response rate). Responses from four clinicians were excluded from the data analysis because they only answered the demographic subset of the survey questions. Most to all were satisfied with the comprehensive nature and utility of the DAGSY report and agreed that DAGSY meets an unmet clinical need and contributes to the quality of care for children with genetic risk variants for NPD (see Fig. 3b). Clinicians were somewhat less satisfied with elements of the clinical process (wait times for the family to be seen and timeliness of the DAGSY report). Suggestions for improvement included increased clinical resources to expand services and provide quicker access to assessments, offering longitudinal follow-up for some children, and better assistance and advocacy for access to school-based and mental health services.

## Discussion

While the potential benefits of diagnosing a genetic etiology for children with NPD are well documented, clinical knowledge of genetically mediated vulnerability for NPD identified prior to the manifestation of developmental or behavioral concerns is still in its infancy. Many parents may feel unprepared [46] and struggle to cope with this “shadow of uncertainty” about their children’s future [47], requiring guidance regarding monitoring their child without becoming overly anxious and overinterpreting each behavior as the “first sign” of mental illness [48]. This is also a challenge for many mental health clinicians who are accustomed to diagnosis and management of concerns and symptoms once they emerge [8]; diagnostic approaches and interventions for children with genetically mediated NPD vulnerability are currently not part of mental health training [49]. The DAGSY clinic was initiated in an attempt to begin to address these largely unmet needs while also establishing a framework for research.

### Specific characteristics of the clinical approach

A distinguishing feature of DAGSY is that NPD symptoms do not have to be present for the child to be referred for evaluation; the vulnerability for NPD conferred by the genetic variant is sufficient. This was the case for approximately one-fourth of children in our cohort whose referrals were exclusively motivated by genetic risk, with this proportion reaching almost two-thirds in those five years or younger at the time of assessment. This is relevant because the early identification of genetically mediated vulnerability for NPD comes with opportunities for preventative medicine, such as parent-mediated therapy to improve social and communication skills [50] and early behavioral intervention [51]. Ultimately, DAGSY strives to take genetically mediated NPD vulnerability as a starting point (“genetic-diagnosis-first”) and provide assessment-based recommendations to mitigate that risk. From this prevention perspective, the goal is to see children as early as possible, preferably before the manifestation of NPD symptoms. Since the start of our clinic, the proportion of children referred based on genetic vulnerability alone, rather than the presence of NPD symptoms, has increased from 15.3% in the first to 31.5% in the last year.

Geneticists and genetic counselors play a key role in informing parents about their child’s genetic variant and NPD vulnerability and are therefore optimally placed to make referrals to mental health professionals [52]. Accordingly, the majority of children seen in DAGSY were referred by geneticists. The high rates of new NPD diagnoses (over three quarters of children received a new NPD diagnosis at DAGSY), along with the relatively advanced average age at diagnosis for ASD (10 years) and ID (11.3 years), strongly suggest that timely diagnosis of NPD is an unmet need in this population. Regarding ID, only about half of the new diagnoses were updates to a previous global or developmental delay diagnosis, typically provisionally given to very young children. For ASD, the mean age at diagnosis in several US and UK studies is between four and five years [53, 54], more than two times younger than our cohort, again underscoring the deficiency of adequate and timely diagnosis in this population. Importantly, a delay of accurately diagnosing neurodevelopmental disorders can negatively impact children’s eligibility for early interventions and educational supports that have the potential to improve their functional outcomes. One possible explanation for the delay in this group, warranting further investigation, is that a genetic diagnosis and frequent medical multi-morbidity in some way “overshadows” the recognition of core symptoms of other relevant neurodevelopmental conditions [55, 56]. The early detection based on genetic risk opens up unprecedented opportunities for early intervention trials. Currently, funding is sought for such studies; however it is important to note that assessment and diagnostic clarification can have, in and by themselves, beneficial impacts [57]. The early diagnosis of an NPD also helps parents to advocate for the right support in school. In the case of ASD or ID, it can provide access to government-funded services and, in some cases, financial support. Even in the absence of such supports, the timely diagnosis of cognitive impairment or learning difficulties allows for an adjustment of the academic expectations, preventing undue stress. Accordingly, the most frequent recommendation to come out of DAGSY evaluations was directed toward parents and schools in the form of concrete strategies to deal with current behavioral and learning issues. Proactive approaches to minimize the child’s stress and anxiety were also highlighted as a way to manage or mitigate future mental health risks, a topic that healthcare providers may not always address adequately with parents [48].

An important aspect that is easily overlooked is the mental and emotional well-being of caregivers and other family members in light of the burden of care around the child with the genetic condition [58]. Concrete recommendations from DAGSY in this regard often involved rallying support from additional sources and/or respite services. Finally, for some individuals, especially those with pathogenic variants associated with later onset psychiatric conditions, DAGSY recommendations included repeated assessments to evaluate possible changes to children’s cognitive trajectories and the emergence of mental health concerns over time.

One significant limitation of DAGSY is its emphasis on providing comprehensive one-time consultations at the expense of not offering long-term follow-up. Consequently, the responsibility for locating resources and coordinating referrals to other services and programs often falls on parents and primary care providers, increasing their already heavy burden and as such may be formulated as another area of a minimally met need of this population. Responding to the emotional support needs of parents and siblings of children with genetic risk variants [59, 60] and providing them with ongoing access to proactive and developmentally sensitive information are recognized as another resource limitation of DAGSY. Limitations of this study include a possible overrepresentation of children with 22q11DS, among the most common genetic risk variants associated with NPD. We cannot exclude that children with a diagnosis of well-established genetic disorders associated with NPDs, such as Down syndrome or Williams syndrome, may already be followed by clinics specializing in these disorders. As a result, they may be underrepresented in our DAGSY population. Also, most referrals to DAGSY were from clinical genetics and genetic disorder-specific clinics within the hospital, where there may be greater awareness of the need for assessment and surveillance for NPD. Finally, limited demographic information was available for the participants in the study.

### DAGSY as a platform for research

In the field of psychiatry genetics, one of the key challenges is enrolling sufficient numbers of individuals to achieve the statistical power needed for genotype-phenotype analyses. This challenge is arguably even more pronounced in the context of rare NPD-related pathogenic variants, thus necessitating large-scale collaborations, as advocated by the Genes to Mental Health network (G2MH; [8]). By implementing a comprehensive, standardized assessment encompassing key phenotypic domains of cognition, development, and behavior, DAGSY functions as a platform facilitating collaborative studies.

## Conclusion

Due to advances in genetic testing technology and clinical uptake, a growing number of children are being identified with genetic variants that increase their vulnerability for an NPD [8]. Responding to the needs of this population requires bringing together psychiatry, psychology, and genetics [61]. There are specialized clinics that provide comprehensive care to children with genetic variants such as those underlying Down syndrome, 22q11DS, Fragile X, Angelman and Prader-Willi syndromes, often embedding psychiatry and/or psychology within a broader service model [62–65]. Other approaches have involved integrating genetics into an outpatient psychiatric clinic for children with autism and/or ID to facilitate genetic evaluations and follow-up [66, 67]. However, to the best of our knowledge, the DAGSY Clinic at SickKids is the first interdisciplinary clinic offering an integrated assessment by clinicians with expertise across psychiatry, development, cognition and genetics. This interdisciplinary approach may facilitate the identification of causal relations between the different domains and ultimately prove to be more efficient and cost effective, cutting down on the number of appointments needed to see different professionals [68].

In summary, DAGSY begins to address the unmet needs of a growing population of families with a child with a substantial vulnerability for a range of NPDs, mediated by genetic vulnerability variants identified very early in life. Increasingly, genetically mediated vulnerability needs to be taken into account in diagnostic assessments and the planning of individualized clinical care pathways. This emerging knowledge comes with new challenges as well as opportunities for precision child health approaches and, possibly, early intervention strategies. By sharing operational details and preliminary outcomes of the DAGSY clinic, we hope it can serve as a model which addresses both clinical and research needs of children at genetic risk for neurodevelopmental and psychiatric conditions.

### Electronic supplementary material

Below is the link to the electronic supplementary material.

Supplementary Material 1

## Data Availability

The survey datasets used and/or analyzed during the current study are available from the corresponding author on reasonable request.

## Footnotes

1 We acknowledge that the concept of risk in relation to genes and NPDs is sensitive, with some stakeholders opposing the terminology. Similarly, discussions in the community are ongoing regarding various aspects of autism-related language preferences [9]. Here, we will use the term ASD, and “vulnerability” and “likelihood” whenever possible, and sometimes “risk” if required for clarity.

## Abbreviations

DAGSY: Developmental Assessment of Genetically Susceptible Youth
NPD: Neurodevelopmental psychiatric disorder
ASD: Autism Spectrum Disorder
ADHD: Attention Deficit Hyperactivity Disorder
ID: Intellectual Disability
GDD: Global Developmental Delay
LD: Learning Disability
22q11DS: 22q11.2 Deletion Syndrome
REDCap: Research Electronic Data Capture
