# Supplementary material for: An integrated clinical approach to children at genetic risk for neurodevelopmental and psychiatric conditions: interdisciplinary collaboration and research infrastructure
Source: J Neurodev Disord. 2024 Jul 5;16:37. doi: 10.1186/s11689-024-09552-x (PMC11229023; doi:10.1186/s11689-024-09552-x)
Supplement: Supplementary file 1 — Supplementary Material 1 [file 11689_2024_9552_MOESM1_ESM.docx]

**Supplement 1**: List of assessment measures used in DAGSY clinical evaluations

| Clinical measures and indications | Age Range |
| --- | --- |
| **Psychiatric Assessment** |  |
| Kiddie Schedule for Affective Disorders and Schizophrenia for School Aged Children – Present and Lifetime (K-SADS-PL)^1^ | 6 years to 18 years, with adaptations for children under the age of 6 and those with lower developmental levels and verbal abilities |
| **Cognitive Functioning** |  |
| Mullen Scales of Early Learning (MSEL)^2^ | Birth to 68 months |
| Wechsler Preschool and Primary Scale of Intelligence, Fourth Edition: Canadian (WPPSI-IV)^3^ | 2 years, 6 months to 7 years, 7 months |
| Wechsler Intelligence Scale for Children, Fifth Edition: Canadian (WISC-V)^4^ | 6 years to 16 years, 11 months |
| Wechsler Adult Intelligence Scale, Fourth Edition (WAIS-IV)^5^ | 16 years to 90 years, 11 months |
| Wechsler Abbreviated Scale of Intelligence, Second Edition (WASI-II)^6^ | 6 years to 90 years, 11 months |
| **Language Assessment** |  |
| Oral and Written Language Scales, Second Edition (OWLS-II)^7^ | 3 years to 21 years, 11 months |
| Peabody Picture Vocabulary Test, Fifth Edition (PPVT-5)^8^ | 2 years, 6 months to 90+ years |
| **Academic Achievement** |  |
| Wide Range Achievement Test, Fifth Edition (WRAT-5)^9^ | 5 years to 85+ years |
| **Social Communication Assessment** |  |
| Autism Diagnostic Observation Schedule, Second Edition (ADOS-2)^10^ | 12 months to adult |
| Autism Diagnostic Interview, Revised (ADI-R)^11^ | 2 years to adult |
| **Adaptive Functioning** |  |
| Vineland Adaptive Behavior Scales, Third Edition (VABS-3)^12^ | Birth to adult |
| **Behavior and Social Ability** |  |
| ASEBA Child Behavior Checklist – Parent Form (CBCL)^13,14^ | 1.5 years to 18 years |
| Social Responsiveness Scale, Second Edition (SRS-2)^15^ | 2 years, 5 months to 18 years |
| SNAP-1V 26^16^ | 6-18 years |
| Repetitive Behavior Scale (RBS)^17^ | 3 years - 48+ years |

References

1. Kaufman J, Birmaher B, Brent D, et al. Schedule for Affective Disorders and Schizophrenia for School-Age Children-Present and Lifetime Version (K-SADS-PL): initial reliability and validity data. *J Am Acad Child Adolesc Psychiatry*. 1997;36(7):980-988. doi:10.1097/00004583-199707000-00021

2. Mullen E. Mullen Scales of Early Learning. Western Psychological Services, 1995.

3. Wechsler D. Wechsler Preschool and Primary Scale of Intelligence, Fourth Edition: Canadian. NCS Pearson, 2012.

4. Wechsler D. Wechsler Intelligence Scale for Children, Fifth Edition: Canadian. Pearson, 2014.

5. Wechsler D. Wechsler Adult Intelligence Scale, Fourth Edition: Canadian. Pearson, 2008.

6. Wechsler D. Wechsler Abbreviated Scale of Intelligence, Second Edition. NCS Pearson, 2011.

7. Carrow-Woolfork E. Oral and Written Language Scales, Second Edition. Western Psychological Services, 2011.

8. Dunn D. Peabody Picture Vocabulary Test, Fifth Edition. Pearson, 2018.

9. Wilkinson G, Robertson G. Wide Range Achievement Test, Fifth Edition. Pearson, 2017.

10. Lord C, Rutter M, DiLavore P, Risi S, Gotham K, Bishop S. Autism Diagnostic Observation Schedule, Second Edition. Western Psychological Services, 2012.

11. Rutter M, Le Couteur A, Lord C. Autism Diagnostic Interview-Revised. Western Psychological Services, 2003.

12. Sparrow S, Cicchetti D, Saulnier C. Vineland Adaptive Behavior Scales, Third Edition. NCS Pearson, 2016.

13. Achenbach T, Rescorla L. Child Behavior Checklist for Ages 1 1/2-5. ASEBA, University of Vermont, 2000.

14. Achenbach T. Child Behavior Checklist for Ages 6-18. ASEBA, University of Vermont, 2001.

15. Constantino J. Social Responsiveness Scale, Second Edition. Western Psychological Services, 2012.

16. Swanson J. SNAP-IV 26 Teacher and Parent Rating Scale. University of California, Irvine, 1981.

17. Lam KSL, Aman MG. The Repetitive Behavior Scale-Revised: independent validation in individuals with autism spectrum disorders. *J Autism Dev Disord*. 2007;37(5):855-866. doi:10.1007/s10803-006-0213-z

**Supplement 2 :** Caregiver and clinician survey

**Family survey**

Part A. Perceived benefits of DAGSY evaluation

Indicate whether you agree or disagree with each statement.

| Survey item | Strongly agree  🞏 | Agree  🞏 | Neutral  🞏 | Disagree  🞏 | Strongly disagree  🞏 | N/A  🞏 |
| --- | --- | --- | --- | --- | --- | --- |
| 1. I have a better understanding of my child’s genetic vulnerability for developing a neurodevelopmental or mental health disorder. |  |  |  |  |  |  |
| 1. I have a better understanding of my child’s existing neurodevelopmental or mental health disorder. |  |  |  |  |  |  |
| 1. I have a better understanding of my child’s strengths and weaknesses. |  |  |  |  |  |  |
| 1. I have a better understanding of the services and supports at school that can help my child (select N/A if child is not in school). |  |  |  |  |  |  |
| 1. I have a better understanding of the services, programs or therapies outside of school that can help my child. |  |  |  |  |  |  |
| 1. I learned about behavioral strategies I can use to help my child. |  |  |  |  |  |  |
| 1. I learned about medications or medication changes to help my child. |  |  |  |  |  |  |
| 1. I feel better equipped to deal with my child’s challenges. |  |  |  |  |  |  |
| 1. I feel more encouraged for my child’s future. |  |  |  |  |  |  |

I have shared the DAGSY report with my child’s school. 🞏 No 🞏 Yes

If yes, did the report lead to changes at school? 🞏 No 🞏 Yes. If yes, please describe the change [open field text]

I have tried to implement recommendations from the report. 🞏 No 🞏 Yes. If yes, what recommendations were helpful?[(open field text]

Part B. Satisfaction with DAGSY services

Indicate whether you agree or disagree with each statement.

| Survey item | Strongly agree  🞏 | Agree  🞏 | Neutral  🞏 | Disagree  🞏 | Strongly disagree  🞏 | N/A  🞏 |
| --- | --- | --- | --- | --- | --- | --- |
| I am satisfied with my experience with the DAGSY clinic. |  |  |  |  |  |  |
| I am satisfied with the quality of care provided by the DAGSY clinic. |  |  |  |  |  |  |
| I am satisfied with the wait time to be seen by the DAGSY clinic. |  |  |  |  |  |  |
| I am satisfied with my initial meeting with the DAGSY clinic to talk about my child and any concerns I had. |  |  |  |  |  |  |
| I am satisfied with the feedback meeting with the DAGSY clinic to discuss the results and recommendations. |  |  |  |  |  |  |
| I am satisfied with the information in the written report from the DAGSY clinic. |  |  |  |  |  |  |

What do you appreciate most about the DAGSY clinic? [open field text]

Do you have any suggestions to improve the DAGSY clinic? [open field text]

**Clinician survey**

The following statements are related to your experience with and views of the DAGSY clinic. Indicate whether you agree or disagree with each statement.

| Survey item | Strongly agree  🞏 | Agree  🞏 | Neutral  🞏 | Disagree  🞏 | Strongly disagree  🞏 | N/A  🞏 |
| --- | --- | --- | --- | --- | --- | --- |
| Criteria for making a referral to the DAGSY clinic were clear |  |  |  |  |  |  |
| Wait time for my patient to be seen by the DAGSY clinic was reasonable. |  |  |  |  |  |  |
| Report from the DAGSY clinic was received in a reasonable time. |  |  |  |  |  |  |
| Report from the DAGSY clinic addressed my referral question. |  |  |  |  |  |  |
| Report from the DAGSY clinic was comprehensive. |  |  |  |  |  |  |
| Report from the DAGSY clinic contained useful information for me. |  |  |  |  |  |  |
| Report from the DAGSY clinic contained useful information for the family. |  |  |  |  |  |  |
| DAGSY meets a clinical demand that previously was not sufficiently addressed |  |  |  |  |  |  |
| DAGSY clinic contributes to improved overall quality of care for children with genetic risk variants for developing a neurodevelopmental or mental health disorder. |  |  |  |  |  |  |

What do you appreciate about the DAGSY clinic? [open field text]

Is there anything you would recommend to improve the DAGSY clinic? [open field text]

**Supplement 3:** Detailed description of the DAGSY clinical process

After a brief introduction, the psychiatrist or fellow discusses the purpose and goals of DAGSY with the parent and child. Typically, this also includes a brief review of the child’s specific genetic variant and what is known about its possible association with behavioral, learning and mental health challenges, utilizing information that was gathered in advance of the clinic appointment. Although most parents already have this knowledge, taking time for this review can be very valuable since they often share past experiences with medical professionals (in general medicine or mental health) with no or limited *a priori* knowledge about the genetic disorder of their child.

The formal assessment starts with a clinical interview and observation of the child with a parent (or without, if so desired by older youth). Subsequently, the interview is continued with parents only, while the child simultaneously undergoes standardized cognitive, language and academic testing by a psychometrist in a separate space. A standardized clinical protocol is used, which covers psychiatric symptoms, cognitive (IQ) and adaptive functioning, language, academic skills (for school-aged children), social communication and behavior (see Supplement 1 for a list of assessment measures). The interviews are conducted in a manner that permits completing the K-SADS-PL^1^ for psychiatric symptoms. When needed, questions are formulated for younger children and children with lower developmental levels and verbal abilities. The selection of some of the formal measures (e.g., cognitive assessments, ADOS-2) is adjusted according to children’s age, verbal abilities or non-verbal mental age, following test-specific guidelines.

Formal psychological assessment requires between 3-5 hours, with breaks being provided to the child as needed. When possible, parents often use the time while waiting for the psychological assessment to be finalized to complete the behavioral questionnaires that are part of the DAGSY assessment. The in-person intake is followed up with two phone interviews, one with parents to collect more in-depth information about their child’s early social and communication development and adaptive functioning, and one with the child’s teacher to obtain information about the school placement, specialized supports, academic progress and general observations of classroom behavior. A single comprehensive report is generated which strives to integrate psychiatric and psychological findings along with the child's specific genetic and environmental context. Diagnostic conclusions and recommendations are formulated and discussed with parents in a separate feedback session, typically as a virtual appointment. This marks the end of the clinical service provided by DAGSY; at this time, parents are also informed about potential research opportunities. Parents receive a copy of the report along with the referring clinician and other health care providers within the child’s circle of care.

References

1. Kaufman J, Birmaher B, Brent D, et al. Schedule for Affective Disorders and Schizophrenia for School-Age Children-Present and Lifetime Version (K-SADS-PL): initial reliability and validity data. *J Am Acad Child Adolesc Psychiatry*. 1997;36(7):980-988. doi:10.1097/00004583-199707000-00021
